# Dysconnectivity of Multiple Resting-State Networks Associated With Higher-Order Functions in Sensorineural Hearing Loss

**DOI:** 10.3389/fnins.2019.00055

**Published:** 2019-02-05

**Authors:** Ying Luan, Congxiao Wang, Yun Jiao, Tianyu Tang, Jian Zhang, Gao-Jun Teng

**Affiliations:** Jiangsu Key Laboratory of Molecular and Functional Imaging, Department of Radiology, Zhongda Hospital, Medical School of Southeast University, Nanjing, China

**Keywords:** sensorineural hearing loss, resting-state network, functional reorganization, independent component analysis, functional connectivity, cognition, emotion

## Abstract

**Objects***:* Sensorineural hearing loss (SNHL) involves wide-ranging functional reorganization, and is associated with accumulating risk of cognitive and emotional dysfunction. The coordination of multiple functional networks supports normal brain functions. Here, we aimed to evaluate the functional connectivity (FC) patterns involving multiple resting-state networks (RSNs), and the correlations between the functional remodeling of RSNs and the potential cognitive or emotional impairments in SNHL.

**Methods***:* Thirty long-term bilateral SNHL patients and 39 well-matched healthy controls were recruited for assessment of resting-state functional magnetic resonance imaging and neuropsychological tests.

**Results:** Using independent component analysis, 11 RSNs were identified. Relative to the healthy controls, patients with SNHL presented apparent abnormalities of intra-network FC involving right frontoparietal network, posterior temporal network, and sensory motor network. Disrupted between-network FC was also revealed in the SNHL patients across both higher-order cognitive control networks and multiple sensory networks. Eight of the eleven RSNs showed altered functional synchronization using a seed network to whole brain FC method, particularly in the ventromedial prefrontal cortex. In addition, these functional abnormalities were correlated with cognition- and emotion-related performances.

**Interpretations:** These findings supported our hypotheses that long-term SNHL involves notable dysconnectivity of multiple RSNs. Our study provides important insights into the pathophysiological mechanisms of SNHL, and sheds lights on the neural substrates underlying the possible cognitive and emotional dysfunctions following SNHL.

## Introduction

Approximately 500 million individuals suffer from HL worldwide. Growing evidence indicates that wide-ranging functional remodeling is involved in the neural mechanisms of HL. Additionally, cognitive and emotional impairments are another consequence of HL, although the causal relationships and underlying mechanisms remain unclear (Smith and Pichora-Fuller, 2015; Contrera et al., 2017). To date, no treatment has been proven highly specific (Schreiber et al., 2010).

The advance of neuroimaging techniques allows studying the central nervous system in an efficient and non-invasive way. fMRI based on the BOLD signal provides a robust method to assess the functional significance following multiple neuropsychiatric disorders. Abnormalities of the central brain associated with HL have previously been documented using multiple neuroimaging methods. MRS results revealed lower GABA concentration in Heschl’s gyrus in presbycusis patients (Gao et al., 2015). Structural neuroimaging research has demonstrated that peripheral HL contributes to the volume decline in temporal area in HL (Lin et al., 2014). The resting-state study reported altered spontaneous neural activity, and intra- or inter-regional functional synchronization within the auditory brain in HL (Li et al., 2013).

Although the auditory area was the most focused area in previous studies, some previous evidence indicates that SNHL is involved in multiple brain networks and systems. Hearing deprivation can enhance the perceptual performance in the intact sensory modalities (Levänen and Hamdorf, 2001; Dye et al., 2009). Human epidemiological studies revealed the associations between HL and accelerated cognitive decline with profound influences on memory (Smith and Pichora-Fuller, 2015), attention (Guerreiro and Van Gerven, 2017), and executive functions (Lin et al., 2011). HL is even implicated in promote the development of dementia (Gurgel et al., 2014). HL is also linked to some emotional impairments, such as depression (Rutherford et al., 2018) and anxiety (Contrera et al., 2017). Several higher-order functional networks were speculated to participate in the functional reorganization in SNHL. For example, alterations of nodal topological properties and FC within the DMN were reported in long-term unilateral SNHL patients (Zhang et al., 2015). Apart from the DMN, the DAN, VN, and AN were also suggested to be involved in HL (Husain et al., 2014; Zhang et al., 2018). These previous findings indicate that HL also has great impacts on the non-auditory systems, including low-level sensory systems and higher-order systems. However, the exact configuration of FC among multiple networks involved in the SNHL remains unclear.

The human brain is a complex system with multi-network interactions. The optimal balance between specialization and integration of the networks enables the human brain to perform a variety of tasks or functions correctly. Hence, it is difficult to explain neural mechanisms underlying HL limited by a particular functional network in isolation. The examinations of intra-network FC could shed lights on the segregation of brain functions, while investigating the network to the whole brain and studying the inter-network FC enhances the understanding of the integration of brain functions. The combination of these approaches could provide important insights into the pathophysiological substrates of HL.

A major limitation of previous studies on HL with the focus on RSNs was that they focused on a certain RSN and ignored the coordinate influence of multiple RSNs on the functional reorganization and the HL-related cognitive or emotional impairments. To overcome these limitations, we assessed the functional alterations in SNHL using ICA to validate the involvement of multiple networks in the functional remodeling related to SNHL, and correlate these multi-network functional modifications with neuropsychological performances to reveal the basis of the possible negative cognitive or emotional outcomes. ICA is a data-driven method to decompose the resting-state fMRI data into several ICs. It is an objective and straightforward way to evaluate the synchronization within multiple networks and the interactions among these networks. Different from other FC analysis, ICA could efficiently separate physiological artifacts from RSNs signals and need no prior assumptions.

This study aimed to validate the following hypotheses, first, long-term bilateral SNHL contributes to distinct alterations of the network to the whole brain, intra- and inter-network FC involving multiple RSNs; second, these changes are associated with neuropsychological performances related to higher-order functions in SNHL patients.

## Materials and Methods

### Participants

Thirty chronic bilateral SNHL patients and 39 normal-hearing controls were recruited into this research. The two groups were well-matched in terms of age, sex and education, and the racial category of all the participants was Chinese Han. The participants in the SNHL group were all right-handed, 19 were male, and 11 were female. The ages ranged from 31 to 69 years. Twenty nine of the SNHL patients did not have a clear etiology, one of them had an etiology of the ototoxic drug. The participants in the control group were all right-handed, 23 were male, and 16 were female. The ages ranged from 32 to 69 years. The detailed demographical information was presented in Table 1. The criteria of the recruitment for the SNHL patients were as follows: (1) age range: 20–70 years; (2) clinically diagnosed SNHL with a disease duration above 3 years; (3) post-lingual HL; (4) bilateral HL with mean hearing thresholds above 25 dB HL for both ears. The criteria of exclusion for both control and SNHL group were as follows: clinically diagnosed Meniere’s disease and acoustic neuroma, self-reported hyperacusis and tinnitus, a clinical history of head injury, cancer, stroke or otologic surgery, poorly controlled diabetes and hypertension, anemia, seizures, multiple sclerosis, Parkinson’s disease, Alzheimer’s disease, depression, schizophrenia and other neurological or psychiatric diseases. This work was proved by the Ethics Committee of Affiliated Zhongda Hospital of Southeast University, and the written informed consent was obtained from each of the participants before the experiments. This research was conducted referring to the Declaration of Helsinki.

**Table 1:** Demographic, clinical, and neuropsychological characteristics of the SNHL patients and healthy controls.

	SNHL patients (*n* = 30)		Healthy controls (*n* = 39)		*p*-Value
	Mean ± SD	Median (interquartile range)	Mean ± SD	Median (interquartile range)	
Age (years)	54.17 ± 10.02		54.50 ± 8.05		0.879
Gender (M/F)	19/11		23/16		0.649
Education (years)	11.37 ± 3.00		11.76 ± 3.29		0.609
Handedness (R/L)	30/0		39/0		
PTA of right ear (dB HL)		38.13 (26.00-69.06)	18.10 ± 5.19	18.75 (14.69-22.81)	**<0⋅0001^∗∗∗∗^**
PTA of left ear (dB HL)		40 (31.25-67.81)	17.76 ± 4.86	18.13 (12.50-22.5)	**<0⋅0001^∗∗∗∗^**
Averaged PTA of both ears (dB HL)		4.063 (30.00-67.19)	17.76 ± 4.86	18.75 (13.44-21.88)	**<0⋅0001^∗∗∗∗^**
**Neuropsychological tests**					
MMSE		30 (29-30)		30 (30-30)	0.165
AVLT	17.00 ± 3.61		17.43 ± 5.46		0.724
AVLT-5	6.07 ± 2.15		6.93 ± 1.952		0.114
AVLT-20		6 (5-7)		7 (5-8)	0.068
SDMT	37.37 ± 12.31		40.27 ± 9.57		0.335
HAM-D		5 (3-8)		4 (2-5)	0.167
SAS	31.17 ± 7.43		27.39 ± 4.10		**0.021^∗^**

*All the data were presented as Mean ± SD. ^∗^p < 0.05, ^∗∗∗∗^p < 0.0001. M/F, male/female; R/L, right/left; PTA, pure-tone average from 0.5 to 4 kHz; MMSE, Mini Mental State Examination; AVLT, Auditory Verbal Learning Test; AVLT-5, Auditory Verbal Learning Test 5-min recall; AVLT-20, Auditory Verbal Learning Test 20-min recall; SDMT, Symbol Digit Modalities Test; HAM-D, Hamilton Depression Rating Scale; SAS, Self-Rating Anxiety Scale. The bold p-values indicate statistical significance (p < 0.05).*

### Audiological Assessment

**FIGURE 1:**
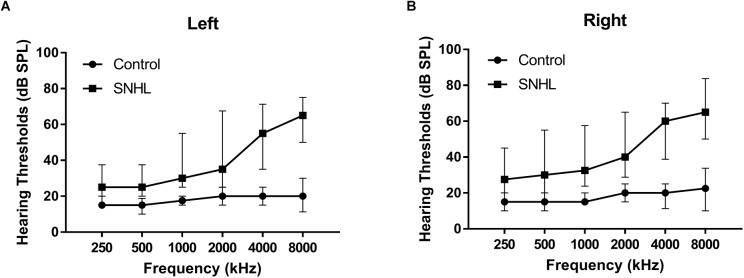
The hearing thresholds of **(A)** right side and **(B)** left side at 250, 500, 1000, 2000, 4000, and 8000 Hz in the SNHL and HC groups. Date are shown as mean ± SD.

The hearing thresholds were measured through pure tone audiometry at the frequencies from 250, 500, 1000, 2000, 4000, and 8000 Hz using a GSI-61 audiometer. The acoustic immittance test was performed to exclude the HL resulting from the conductive disturbance. The speech-frequency pure-tone average (PTA) of air-conduction thresholds at the frequencies of 500, 1000, 2000, and 4000 Hz was calculated for each side of ears (Agrawal et al., 2008; Gao et al., 2015; Choi and Park, 2017; Zhang et al., 2018). The binaural mean hearing threshold was calculated by averaging the monaural PTA for each participant. In accordance with the World Health Organization (WHO) classification standard (1997), HL was defined as a PTA > 25 dB in either ear. All the participant in the control group had the PTA value < 25 dB HL for each ear. All the participants in the SNHL group had the PTA value > 25 dB HL for each ear (Figure 1 and Table 1).

### Neuropsychological Assessment

All the participant underwent a series of neuropsychological evaluations that covered the relevant cognitive or emotional domains. All tests took approximately 1 h to complete. For the cognitive assessment, the general cognitive state was determined by the MMSE (Galea and Woodward, 2005). The functions of attention, executive control, visual scanning and the speed of information processing were evaluated using the SDMT (Smith, 1982). The episodic memory regarding verbal information was assessed by the AVLT (Schmidt, 1996) comprising an immediate recall, a 5-min delayed recall and a 20-min delayed recall test. For the emotional assessment, the possible depression states were determined via the HMA-D (Hamilton, 1960) and the possible anxiety states were determined via the SAS (Zung, 1971). For each participant, all these neuropsychological evaluations were conducted in the same order by the same researcher.

### MRI Data Acquisition

The MRI data of each participant were all obtained at the Department of Radiology, Affiliated Zhongda Hospital of Southeast University using a Siemens 3.0 T MRI scanner (Siemens, Erlangen, German) with a 12-channel head coil. Soft foam padding was used to alleviate head motion, and earplugs were used to alleviate the noise during scanning. The participants were instructed to keep heads still, eyes closed and not to think about any particular things inside the MRI scanner. Functional raw images were acquired in an interleaved order using a gradient-recalled echo-planar imaging (GRE-RPI) sequence (repetition time 1900 ms, echo time 2.48 ms, flip angle 90.0°, slice number 32, slice thickness 4.0 mm, the field of view 240 mm × 240 mm, matrix 64 × 64, volumes 240). Structural images were acquired for registration and localization of functional images using a high-resolution three-dimensional magnetization-prepared rapid gradient-echo (3D MPRAGE) T1-weighted sequence (repetition time 1900 ms, echo time 2.48 ms, flip angle 9.0°, inversion time 900 ms, slice number 176, slice thickness 1.0 mm, field of view 250 mm × 250 mm, matrix 256 × 256).

### Functional Data Preprocessing

The preprocessing of all the GRE-EPI images for each subject were carried out with the Statistical Parametric Mapping software (SPM8^1^) and the Data Processing and Analysis for Brain Imaging V2.3 (DPABI^2^) toolbox. Preprocessing included the following steps: removal of the first 10 volumes of the total 240 volumes; slice-timing adjustment; realignment for head motion correction (subjects with head motion over 2.0 mm of the maximum translation or 2.0 degrees of axial rotation were excluded), non-linearly normalization by Diffeomorphic Anatomical Registration using Lie Algebra (DARTEL) algorithm using New Segment to a standard template in the MNI space with a resampled voxel size of 3 mm × 3 mm × 3 mm; spatial smoothing with a 6-mm FWHM Gaussian kernel. No participant was excluded in the current study due to excessive head motion.

### Independent Component Analysis

Group ICA (Beckmann et al., 2005) was performed using Group ICA of fMRI Toolbox (GIFT) version 4.0a^3^. This analysis could reveal the spatio-temporal associations across the global human brain. The analysis procedures were conducted in accordance with common methods (Calhoun et al., 2009; Cong et al., 2014). Briefly, the date reduction was employed using a two-step principal component analysis (PCA) to reduce the dimensions of the functional data (Calhoun et al., 2001). Here, we selected a moderate model order of 40 to determine the ICs which were corresponding to both functional and anatomical networks (Schrepf et al., 2018). Component estimates were then performed using an Infomax ICA algorithm (Bell and Sejnowski, 1995), which is a stochastic process. This step was conducted using the ICASSO algorithm, which repeated the ICA analyses 20 times and generate a final set of ICs, to determine the stability and consistency of the ICs (Himberg et al., 2004). The final step is the back reconstruction, which computed the individual spatial maps and time courses of each IC, using a group ICA 3 (GICA3) method, followed by the processing of grouping the individual components and thresholding the group ICA maps (Calhoun et al., 2001). The spatial maps then underwent a z transformation to obtain the z-score maps (subtracting the means and dividing the results by the standard deviations). The z score in each voxel represented the strength of its contribution to the time course of the IC. The RSNs were identified by spatial correlation and visual check with previously published network templates from ICA for further analyses (Beckmann et al., 2005; Smith et al., 2009). After that, 11 of the 40 ICs were identified to be 11 RSNs, which presented higher low-frequency spectral power with the peak activation located in the GM and minimal overlap with the ventricles or WM.

### Intra- and Inter-Network Functional Connectivity Analyses

For each of identified RSNs, we measured the changes in the intra-network FC in the SNHL group when compared with the control group. Specifically, the network mask of each RSN was generated by setting a threshold of z-score above 1.5 to the group mean spatial map of the corresponding IC. The individual spatial z maps of each identified RSN obtained from the back-reconstruction were compared for the intra-network connectivity between two groups in a voxelwise manner. Between-group comparisons of intra-network connectivity were conducted using ANOVA within the corresponding network masks using a GLM with age, sex, education, and head motion parameter included as nuisance covariates with no interest. The threshold was set at *p* < 0.05, determined by the GRF Theory for the multiple comparison correction.

To explore the FC pattern among different functional networks, the temporal correlations among the identified RSNs were performed (Jafri et al., 2008). The procedures of this analysis were briefly described as follows. Individual time courses of each identified RSN were obtained from the back-reconstruction procedure of ICA. The temporal correlation coefficients of time courses from each pair of the 11 RSNs were then calculated and normalized with Fisher’s r-to-z transformation. The 11 × 11 inter-network connectivity matrices of the SNHL and control groups were conducted respectively for between-group comparisons. Between-group differences in the FC for each pair of identified RSNs were compared using ANOVA with age, sex, education, and head motion as nuisance covariates. The statistical significance was considered as a *p*-value < 0.05, corrected by FDR method.

### Seed Network to Whole Brain Connectivity Analysis

This analysis was conducted using DPABI and the Resting-State fMRI Data Analysis Toolkit (REST^4^). Briefly, the WM, cerebro-spinal fluid and the motion parameters were regressed from the data after preprocessed. The linear drifts were removed. A bandpass filter with the frequency window from 0.01 to 0.1 Hz was applied to remove the high-frequency noise of the functional data. The spatial mask of each RSN was considered as the seed for the calculation of seed network to whole brain FC. The voxelwise FC calculation was conducted according to standard methods as previously reported (Fox et al., 2006). The Pearson’s correlation coefficients were computed between the mean time courses of each seed network and each voxel across the brain for each participant. Fisher’s r-to-z transformation was employed to generate individual seed network to whole brain FC z-score maps. Between-group comparisons for each identified RSN were explored by ANOVA using a GLM with age, sex, education, and head motion parameter included as nuisance covariates with no interest. The cluster threshold was set at *p* < 0.05/11 (Bonferroni corrected), determined by the GRF method for the multiple comparison correction.

### Structural Data Analysis

Voxel-based morphometry assessment was employed to calculate the volume of GM and WM (WM) for each participant using the SPM8 toolbox^5^. The T1 weighted images of each subject were segmented into the GM, WM and cerebro-spinal fluid and then non-linearly normalized to standard MNI space. The images after normalization were smoothed with an 8-mm FWHM Gaussian kernel. Global GM and WM volumes of the participants were identified by estimating the segments. Brain parenchyma volumes were computed as the sum of global GM and WM volumes. The between-group differences in GM and WM volumes were also identified by voxelwise analyses via ANOVA using a GLM with age, sex, and education included as nuisance covariates with no interest. The significant difference was determined with a *p*-value threshold < 0.05 corrected by GRF Theory. The ROI based VBM analysis was used to compare the regional GM between two groups where significant functional changes were determined by functional analyses. The ROI was identified from the between-group comparison results of functional measures. The participant-specific GM data within the ROI was exacted from the normalized and smoothed GM images, and then a one-way ANOVA analysis was performed with a significant *p*-value < 0.05.

### Statistical Analysis

All of clinical variables were tested for normal distribution using the Kolmogorov–Smirnov test. Group differences in age, gender, education, mean hearing thresholds and results of neuropsychological tests were determined with a one-way ANOVA, Mann–Whitney *U*-test or chi-square test (SPSS, 19 software, Chicago, IL, United States). When *p* < 0.05, differences were considered significant. Mean values of the functional metrics with significant differences between two groups were extracted and assessed further for correlations with neuropsychological test results in the SNHL group using a partial correlation analysis, controlling for the age, sex, and education. *p* < 0.05 was considered statistically significant.

## Results

### Demographical, Hearing, and Neuropsychological Characteristics

The SNHL group and control group were well-matched in age, sex, and duration of education, and handedness. All the participants were from Han ethnicity. The mean hearing thresholds of left and right side were above 45 dB HL in the SNHL group. There was no significant difference between two sides. The PTA of both sides in the control group were significantly lower than the SNHL group and were within the normal range (<25 dB HL, Figure 1). For the neuropsychological results, the SNHL patients showed significantly increased SAS scores compared to the healthy controls. No significant difference was observed in the MMSE, AVLT, AVLT-5, AVLT-20, SDMT, or HMA-D tests between SNHL group and control group. The detailed demographic, hearing and neuropsychological results were illustrated in Table 1.

### Resting-State Networks

**FIGURE 2:**
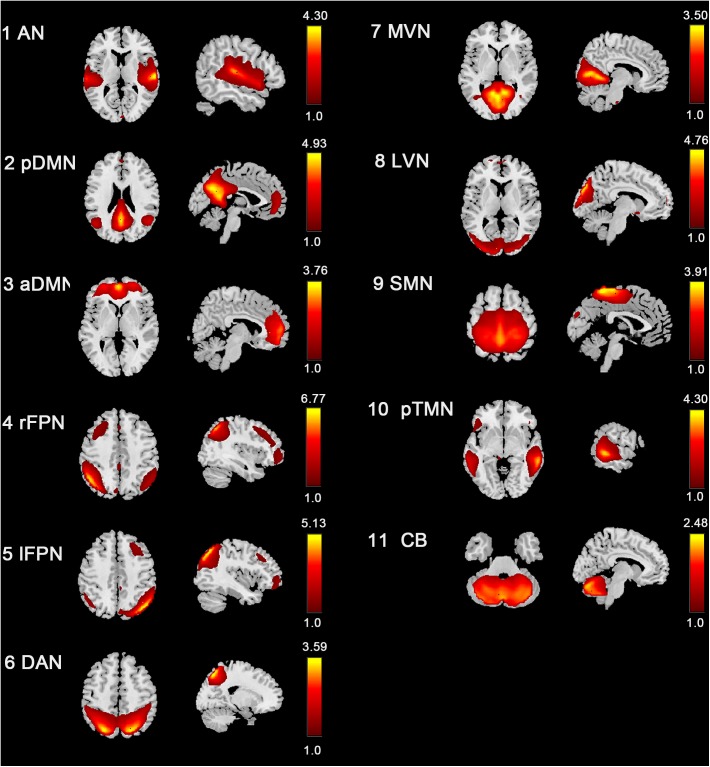
Spatial distribution of 11 intrinsic RSNs determined by ICA. The colormaps represent the *z*-values.

Eleven RSNs were identified out of all 40 ICs by spatial correlations in accordance to previous research (Smith et al., 2009; Peraza et al., 2017; Huang et al., 2018; Zhao et al., 2018). The RSNs included these networks were AN, pDMN, aDMN, rFPN, lFPN, DAN, MVN, LVN, SMN, pTMN, and CB (Figure 2). The AN mainly included bilateral STG. The pDMN mainly concentrated in the PCC, precuneus, and bilateral lateral parietal cortex. The aDMN mostly included ACC and mPFC. The left and right FPN mainly involved the dorsolateral PFC and posterior parietal cortex. The DAN included bilateral inferior parietal sulcus and middle temporal gyrus (MTG). The SMN mainly located in the bilateral precentral and post-central gyrus, and PMC. The MVN and LVN involved medial and lateral part of the occipital lobe. The pTMN located in the posterior temporal lobe bilaterally. And the CB included the most part of the cerebellum.

### Intra-Network Connectivity

**FIGURE 3:**
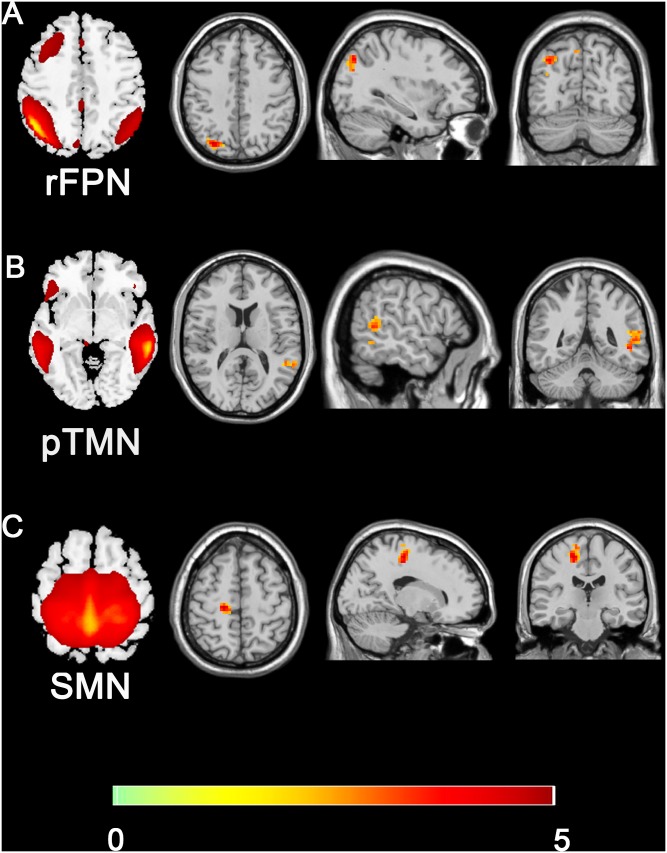
Group difference distributions of intra-network FC between SNHL group and control group **(A–C)**. Warm colors indicate regions with higher intra-network FC in SNHL patients compared with healthy controls (*p* < 0.05, Gaussion Random Field Theory corrected). The colorbar in the bottom shows the t values from the between-group comparison. The schematics of each seed network obtained from ICA were shown in the left of each panel.

**FIGURE 4:**
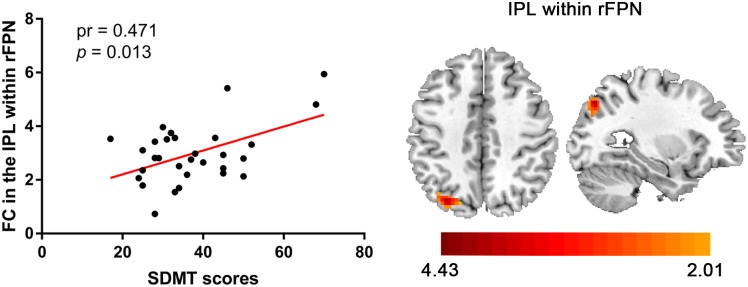
Significantly positive correlation between the altered intra-network FC in IPL within rFPN and SDMT scores in the SNHL group after controlling for the effects of age, sex, and education.

Compared with the control group, the SNHL group presented significant alterations of FC within several RSNs. The FC in the right inferior parietal lobule (IPL) was increased within the rFPN (Figure 3A). The FC in the right PMC was increased within the SMN (Figure 3B). The FC in the left posterior STG and MTG were increased within the pTMN (Figure 3C). The detailed between-group differences in intra-network connectivity were presented in Table 2. Moreover, the changed FC in the right IPL within rFPN presented a significantly positive correlation with the SDMT scores in the SNHL patients, controlling the age, sex, and education (Figure 4).

**Table 2:** Brain regions with significant differences in the intra-network functional connectivity between the SNHL group and control group.

Brain region	Brodmann area	Voxel size	Peak MNI coordinates (mm)			Peak *z*-values
			*X*	*Y*	*Z*	
**rFPN**						
Right inferior parietal lobule	7	85	-30	-75	42	4.4292
**SMN**						
Right premotor cortex	6	82	18	-24	54	4.3972
**pTMN**						
Left superior/middle temporal gyrus	21/22	97	-48	-45	-3	3.8759

*rFPN, right fronto-parietal network; SMN, sensory motor network; pTMN, posterior temporal network.*

### Inter-Network Connectivity

**FIGURE 5:**
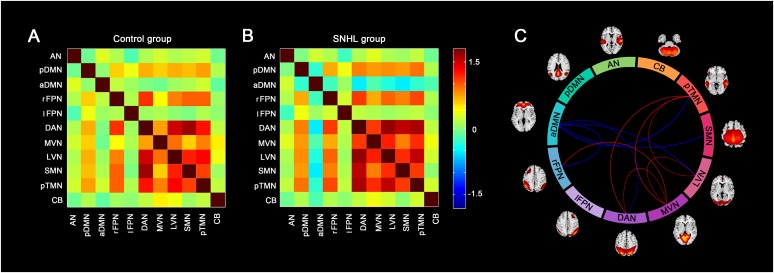
Group difference in the inter-network FC between the SNHL group and control group. **(A,B)** Shows the group averaged FC matrices of the z scores for each pairs of networks derived by ICA in the control group and SNHL group. The heat map next to panel B shows the z-score distribution of group-averaged inter-network FC. **(C)** Show the between-group differences in the inter-network FC. Eleven arcs in the big circle with different colors denote different RSNs used to calculate the FC values. The lines connect the arc pairs represent significant differences in the FC between corresponding RSN pairs (*p* < 0.05, corrected by FDR). Blue lined denote significantly decreased inter-network FC, red lines denote significantly increased inter-network FC in the SNHL group compared to the control group. The brain maps next to the arcs are the schematic representations of the RSNs derived from ICA.

**FIGURE 6:**
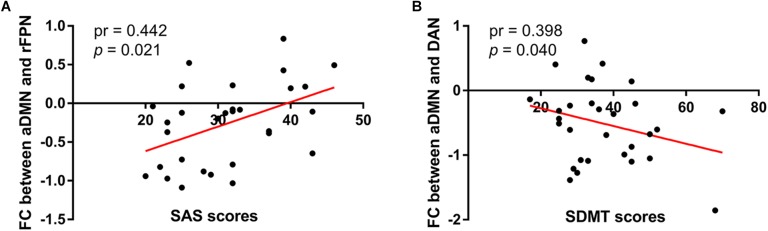
Correlations between magnitudes of changed inter-network FC with neuropsychological results in the SNHL group. The altered inter-network FC between aDMN and lFPN showed significantly positive correlation with SAS scores **(A)**, and the altered FC between aDMN and DAN showed significantly negative correlation with SDMT scores **(B)** in the SNHL group after controlling the effects of age, sex, and education.

Similar between-network connectivity patterns of the control group and SNHL group were illustrated by the averaged FC matrices in Figure 5A,B. Compared with the healthy controls, the SNHL showed significantly declined FC between aDMN and rFPN, DAN, LVN, SMN, and pTMN. In addition, significantly enhanced FC was found between rFPN and MVN, between DAN with MVN and pTMN, between MVN with LVN and pTMN, between LVN with pTMN in the SNHL group (*p* < 0.05, FDR corrected, Figure 5C). Besides, the altered inter-network FC between aDMN and rFPN was positively correlated with the SAS scores, and the altered inter-network FC between aDMN and DAN was negatively correlated with the SDMT scores in the SNHL group controlling the effects of age, sex, and education (Figure 6).

### Seed Network to Whole Brain Connectivity

**FIGURE 7:**
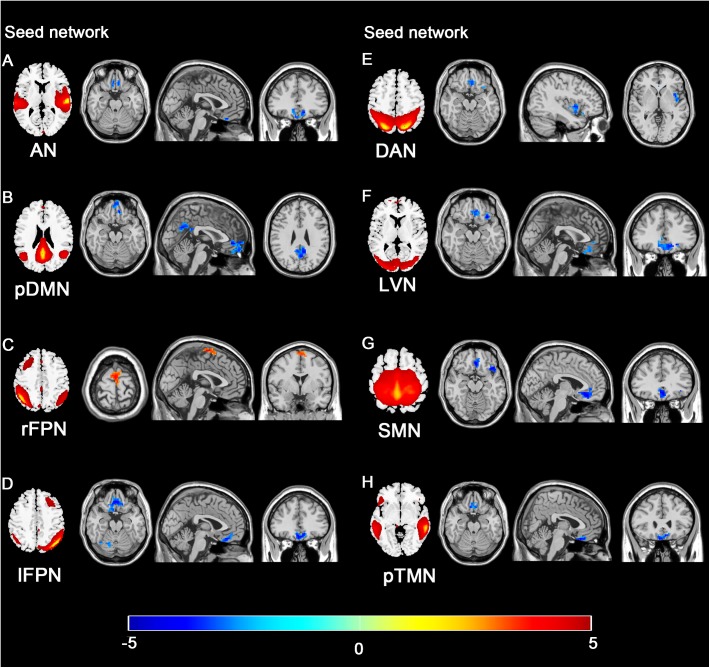
Group difference distributions of seed network to whole-brain FC between SNHL group and control group. Significantly altered FC with AN **(A)**, pDMN **(B)**, rFPN **(C)**, lFPN **(D)**, DAN **(E)**, LVN **(F)**, SMN **(G)**, and pTMN **(H)** are highlighted in the heat bar scales (*p* < 0.05, GRF corrected). The colorbar in the bottom shows the *t*-values obtained from between-group comparison. The schematics of each seed network obtained from ICA were shown in the left of each panel.

The 11 spatial maps of the RSNs identified from ICA were used as the seed to compute the voxel-wise FC across the whole brain. The group differences in the seed network to whole brain connectivity were presented in Figure 7 and Table 3. The FC in the vmPFC with AN, pDMN, lFPN, DAN, LVN, SMN, and pTMN was significantly decreased in the SNHL group. Apart from that, when the seed was set to pDMN, the FC in PCC was significantly decreased in the SNHL group. The FC in the PMC with rFPN was increased. The FC in right cerebellum lobule VI was significantly decreased with seed set at lFPN in the SNHL group. FC between DAN and left insula was significantly suppressed in the SNHL group. The SNHL patients also showed significantly declined FC between SMN and left insula.

**Table 3:** Seed network to whole-brain functional connectivity differences.

Brain region	Brodmann area	Voxel size	Peak MNI coordinates (mm)			Peak *t*-values
			*X*	*Y*	*Z*	
**AN**						
Ventromedial prefrontal cortex	11	111	3	33	-24	-4.2123
**pDMN**						
Ventromedial prefrontal cortex	11	451	-6	57	-3	-4.2921
Posteriors cingulate cortex	31	286	6	-48	9	-4.1017
**rFPN**						
Premotor cortex	6	110	0	-6	72	3.8468
**lFPN**						
Ventromedial prefrontal cortex	11	264	6	27	-27	-4.5769
Right cerebellar lobule VI		203	15	-72	-24	-3.7285
**DAN**						
Ventromedial prefrontal cortex	11	165	-3	30	-15	-3.9780
Left insula	48	115	-39	6	0	-3.4735
**LVN**						
Ventromedial prefrontal cortex	11	270	-33	27	-12	-4.1008
**SMN**						
Ventromedial prefrontal cortex	11	142	-3	30	-12	-3.9315
Left insula	48	162	-39	6	0	-4.5118
**pTMN**						
Ventromedial prefrontal cortex	11	103	-3	21	-27	-4.5023

*AN, auditory network; pDMN, posterior default mode network; rFPN, right fronto-parietal cortex; lFPN, left fronto-parietal cortex; DAN, dorsal attention network; MVN, medial visual network; SMN, sensory motor network; pTMN, posterior temporal network.*

**FIGURE 8:**
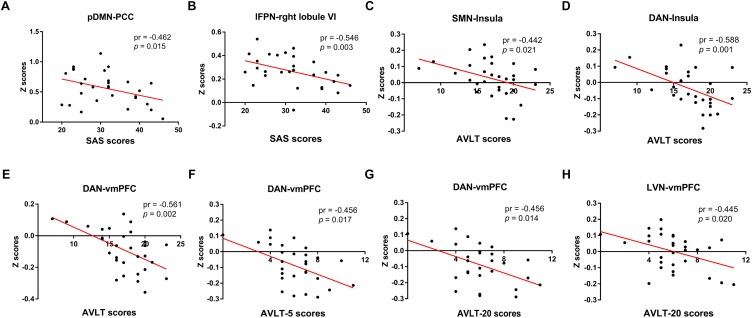
The correlations between altered seed network to whole brain connectivity and neuropsychological results in the SNHL patients, controlling for the age, sex, and education. The FC in PCC with pDMN **(A),** and the FC in right lobule VI with lFPN **(B)** were negatively correlated with the SAS scores. The FC between SMN with insula **(C)**, and between insula and vmPFC with DAN **(D,E)** were negatively correlated with AVLT scores. The FC in the vmPFC with DAN **(F)** was negatively correlated with the AVLT-5 scores. The FC in the vmPFC with DAN and LVN **(G,H)** were negatively correlated with AVLT-20 scores.

Figure 8 illustrated the significant correlations between altered seed network to whole brain FC and neuropsychological results in the SNHL patients after controlling the effects of age, sex, and education. The altered FC in PCC with pDMN (Figure 8A) and the FC in right lobule VI with lFPN (Figure 8B) were negatively correlated with the SAS scores. The FC in the insula with seed set to SMN, and FC in the insula and vmPFC with the seed set to the DAN (Figure 8C–E) had significant negative correlations with AVLT scores in the SNHL patients. The FC between the vmPFC and DAN (Figure 8F) was negatively correlated with the AVLT-5 scores. The FC in the vmPFC with DAN and LVN (Figure 8G,H) also showed significant negative correlations with AVLT-20 scores in the SNHL group, controlling age, sex, and education.

### Structural Results

Global GM, WM, and brain parenchyma volumes showed no obvious between-group changes. There was neither significant difference in GM or WM between the control group and SNHL group in a voxelwise way with the threshold of *p* < 0.05 corrected by GRF. Since vmPFC, particularly within Brodmann area 11 (BA11), presented notable difference in the seed network to whole brain FC between the SNHL group and control group, the GM in the BA11 from the Brodmann Template were compared between the control group and SNHL group. No difference in the GM of vmPFC was observed neither. The statistical data were presented in Table 4.

**Table 4:** Comparisons of the brain volumes between the SNHL patients and healthy controls.

	SNHL patients (*n* = 30)	Healthy controls (*n* = 39)	*t*	*p*-Value
Global GM	619.00 ± 11.02	636.40 ± 12.01	1.040	0.302
Global WM	500.00 ± 9.93	506.00 ± 8.25	0.470	0.640
Brain parenchyma	1119.00 ± 20.64	1142.00 ± 19.69	0.814	0.419
vmPFC GM	21.15 ± 0.38	21.90 ± 0.52	1.114	0.269

*GM, gray matter; WM, white matter; vmPFC, ventromedial prefrontal cortex.*

## Discussion

In this study, we assessed alterations of the seed network to whole brain FC, intra- and inter-network FC, and their correlations with neuropsychological performances related to cognitive and emotional functions in long-term bilateral SNHL patients. Using the ICA method, 11 RSNs were identified from a total of 40 ICs. Three of the 11 RSNs presented abnormalities of within-network FC involving rFPN, pTMN, and SMN. Disrupted between-network FC were also demonstrated in the SNHL patients, mainly concentrating among the aDMN, rPFN, DAN, MVN, LVN, SMN, and pTMN. Eight of the 11 RSNs showed apparent functional coupling changes across the whole brain, particularly in the vmPFC. Additionally, the magnitudes of these functional abnormalities were found to be correlated with neuropsychological results. These findings supported our hypotheses, and enhanced the knowledge around the functional network reconfiguration in long-term SNHL involving both low-level sensory processing networks and high-order cognitive control networks, and the potential mechanism of the cognitive or emotional dysfunction related to SNHL.

The human brain is organized in different intrinsic networks identified by rs-fMRI studies. The regions within the same RSNs present relatively higher functional coupling than those from distinct RSNs (Biswal et al., 1995). Within-network FC supports the specialization of a particular brain function, whereas, the between-network functional communications are essential for implementing the complex functions which require the integration of different functional networks. Our intra- and inter-network connectivity results revealed apparent remodeling of multiple RSNs, including lower-order sensory processing networks and higher-order cognitive control networks.

Hearing loss results in abnormal multiple sensory processing. Cross-modal neuroplasticity, an intrinsic capacity in the brain, is a compensatory mechanism when a specific sensory modality is deprived. The cross-modal reorganization is well-accepted in HL. In cases of deafness, the auditory cortex begins to respond robustly to the somatosensory or visual input (Campbell and Sharma, 2014; Cardon and Sharma, 2018). In the current study, we demonstrated altered between-network synchronization with multiple sensory networks, such as LVN, MVN, SMN, and pTMN. Consistent with previous fMRI findings, HL impacts the FC between sensory cortices. Notably, an increased FC between the auditory cortex and other sensory cortices, such as the visual cortex, has been documented (Puschmann and Thiel, 2017). We demonstrated distinct between-network FC alterations among LVN, MVN and pTMN in SNHL, although no change was found in FC with AN. However, the pTMN, which mostly involves the posterior part of superior and middle temporal cortex, is also associated with auditory processing, as well as visual processing, specifically auditory, and visual language perception (Nakamura et al., 2005). Accumulating evidence suggested the roles of pSTP in auditory spatial information and vocal sound processing (Warren et al., 2005), pSTG/pSTS in coding speech information to both auditory and visual inputs through a multisensory integration mechanism (Park et al., 2018), and the pMTG in semantic object retrieving (Davey et al., 2016). Taken together, the posterior temporal area is involved in specific auditory or visual information processing. In the cases of HL, different part of the temporal lobe presented different intra- and inter-regional synchronization, which was revealed by a recent rs-fMRI study (Li et al., 2013). Based on these notions, the modifications of inter-network FC among VNs and pTMN, together with the intra-network FC in pSTG and pMTG within pTMN reflect the cross-modal functional reorganization following HL. The motor area is essential for speech recognition and can be activated by various classes of sound (Scott et al., 2009). HL patients should exert more effort in speech comprehension and recognition, particularly in noisy environments (Na et al., 2017; Vannson et al., 2017). Additionally, the integration of multi-sensory modalities is essential for providing a unified perception and recognition of the motion and behavior. The response to aurally presented action-sounds in the frontoparietal motor network was documented, including Broca’s area, PMC, intraparietal sulcus, and the inferior parietal region (Lahav et al., 2007). Profound deafness and blindness were found to lead to suppressed resonant motor facilitation on action perception (Alaerts et al., 2011). Hence, the increased intra-network FC in the PMC within the SMN observed in the current study in SNHL patients, together with the increased FC in the PMC with the seed set to rFPN, were proposed to be a compensatory and adaptive mechanism underlying the deficit of such ‘action-listening’ response following HL.

Sensory processing is under the top–down control from frontal and parietal areas linked to sensory attention (Ruff, 2013). Importantly, cross-modal plasticity relies on attentional transitions from the impaired to the retained sensory cortex, requiring higher-order cognitive controls. We found enhanced functional coupling between multiple sensory networks with FPN and DAN, which are involved in the sensory attention control. We also observed increased intra-network FC in the IPL within rFPN in SNHL. Taking together, our findings provide strong evidence about the cross-modal reorganization and top–down control dysfunction of multiple sensory processing after hearing deprivation. Besides, being correlated with the SDMT scores, the hyperconnections in IPL within rFPN also gives a better understanding of the cognitive or executive control impairments potentially rising from SNHL.

Epidemiological investigations have also reported the relationships between HL and abnormal higher-order cognitive control functions, exerting great impacts on memory (Smith and Pichora-Fuller, 2015), executive control (Lin et al., 2011), attention (Guerreiro and Van Gerven, 2017), and language (Halliday et al., 2017). HL can even promote the development of dementia (Gurgel et al., 2014). DMN, an intrinsic network activated without a particular task, plays a crucial role in self-referential mental activity, cognitive control and maintenance of internal and external attention (Leech and Sharp, 2013). Accumulating evidence has demonstrated the involvement of DMN in the functional modifications in HL, which was indicated to be associated with the cognitive dysfunction driven by hearing deprivation. In spite of the wide concern on the role of DMN in HL, the relationship between multiple RSNs with the subdivisions of DMN in HL has rarely been explored so far. The anterior and posterior divisions of DMM interact with each other in a dynamic equilibrium, which preserves the normal use in cognitive processing (Uddin et al., 2009). The dissociation of the sub-networks of DMN has been widely reported in traumatic brain injury, autism and type 2 diabetes mellitus (Starck et al., 2013; Cui et al., 2015). In addition, evidence from recent rs-fMRI studies proposed that the sub-divisions of DMN interact predominantly with several task positive networks, mainly among aDMN, salience network, DAN and rFPN (Di and Biswal, 2014). The current study revealed the dissociated FC pattern of DMN sub-networks in SNHL. Particularly, the apparent inter-network FC alterations of aDMN with DAN and rFPN, together with the decreased seed network to whole brain FC in the PCC which is the core component of pDMN with pDMN, were suggested to be a potential imaging marker to prognosticate the cognitive dysfunction associated with SNHL. Also, emotion-dependent dysfunction of DMN has been demonstrated in several emotional impairments, although the effects of DMN on emotional processing were of relatively little concern (Sreenivas et al., 2012; Ho et al., 2015). The correlations between the inter-network FC with neuropsychological performances supported our assumption, that the dysconnectivity of DMN might be associated with the cognitive or emotional states in SNHL. DMN also participates in the perceptual processing through retrieving the information memory, which is elucidated by recent functional imaging studies (González-García et al., 2018). Based on these notions, this dissociation pattern of FC with aDMN and pDMN observed in this study indicated the involvement of higher-order cognitive networks in the functional reorganization of SNHL and the modified coordination between DMN with both higher-order control networks (i.e., DAN, FPN) and sensory processing networks (i.e., LVN, MVN, SMN, and pTMN) following hearing deprivation.

The seed network to whole brain FC analysis revealed the altered pattern of connectivity with the RSNs across the whole brain. Previous ICA-based research only considered the disease-related intra- or inter-network FC alterations, which could not fully uncover the functional changes involving multiple RSNs. Our results found noticeable differences in seed network to whole brain FC in the SNHL group. Interestingly, the configurations were overlapped in the vmPFC.

The vmPFC is a vital part of prefrontal cortex in mammalian brain, involved in a wide variety of brain functions, such as value-based decision making (Barrash et al., 2000), negative emotional regulation and social cognition (Hiser and Koenigs, 2018). Moreover, the vmPFC has been considered as a core part in the noise canceling system associated with interoceptive-autonomic regulation. Anatomically, the vmPFC has a tight association with the auditory system. The vmPFC gives direct projections to the thalamic reticular nucleus (TRN), including the auditory part of it, which plays a crucial role in the inhibitory modulation of the communication between the auditory thalamus and auditory cortex (Zikopoulos and Barbas, 2006). Under normal conditions, the auditory input originates from the subcortical region of the auditory pathway and then is transmitted to the auditory cortex and vmPFC through the auditory thalamus. Hence, the efficient vmPFC output enables the excitation-inhibition balance maintained during auditory processing. Structural and functional abnormalities of vmPFC might be involved in the deficits in auditory processing, such as tinnitus, which has been widely reported (Leaver et al., 2011, 2016). Considering that HL is usually the peripheral origin of the tinnitus, the question is whether changes in vmPFC are specific to tinnitus. Previous VBM studies reported hearing threshold-related GM volumes and structural changes in the vmPFC in the individuals with HL independent of tinnitus (Boyen et al., 2013; Melcher et al., 2013), inspiring the investigations on the functional alterations of the vmPFC in HL. A recent resting-state FC study revealed changed interregional connectivity between the vmPFC and the brain regions within the PFN and temporal lobe in HL patients (Jung et al., 2017), which is in line with our seed network to whole brain FC results. Our study provided additional evidence that the functional remodeling between the vmPFC and multiple RSNs was involved in the HL with absence of tinnitus. These considerable changes were speculated to be associated with the disturbance of the auditory information transmission in HL. Besides, the vmPFC-amygdala circuit is accepted to play a pivotal role in negative emotion modulation. The inhibitory regulation from the vmPFC exerts hypoactivity on the amygdala, and consequently suppresses the negative affect. The neurocircuitry dysfunctions contribute to a variety of psychiatric and mood disorders (Jalbrzikowski et al., 2017). It has been well-reported that HL is associated with accumulated emotional dysfunction, such as anxiety and depression (Contrera et al., 2017; Rutherford et al., 2018). In accordance with previous investigations, we also found elevated anxiety states in SNHL patients. The functional remodeling with multiple RSNs occurring in the vmPFC was also supposed to be involved in the emotional impairments in SNHL. Nevertheless, the exact nature of the vmPFC-amygdala circuit and the relationship with the emotional deficit in HL should be clarified in future research. In addition, the vmPFC is the core structure of aDMN. Thus, another explanation for the modified FC between vmPFC with multiple RSNs might be to mediate the disrupted inter-network FC with aDMN observed in this work. This hypothesis is supported by previous rs-fMRI research which disclosed the disturbance of functional couplings between the vmPFC, the key node in DMN and the anterior insula, the key node in cognitive control network in HL, suggesting compensatory coordination between these two networks in the perceptual deficit (Wang et al., 2014). We reported the dysconnectivity between aDMN and multiple RSNs. These RSNs were predominantly overlapped with those showing altered seed network to whole brain FC.

Positron emission tomography studies revealed age-dependent resting glucose metabolism and increased stimulated glucose metabolism in the vmPFC in deaf patients, indicating altered neural activity in the vmPFC related to HL (Lee et al., 2007; Herholz et al., 2015). In line with a structural imaging study, we found no change in GM volume of the vmPFC in SNHL, while it was suggested that the vmPFC volume might compensate for the declined peripheral hearing following hearing deprivation (Wong et al., 2010). Although the functional significance of the vmPFC related to HL has rarely been investigated previously, given the intrinsic associations of function and anatomy with the auditory system, it is still plausible that the functional modification occurred in the vmPFC following the reduction of auditory input to the auditory pathway. Our findings expand the understanding of the brain functional alterations involving both low-level sensory processing networks and higher-order cognitive control networks in SNHL, particularly emphasizing the significance in the prefrontal area.

Several other brain regions showed changed functional coupling with the RSNs, including cerebellum and insula. Traditionally, the cerebellum is thought to be involved in the motor balance and physical coordination. Nevertheless, anatomical and functional studies have revealed segregated cerebral-cerebellar circuits in non-human primates (Kelly and Strick, 2003; Krienen and Buckner, 2009), particularly between the frontoparietal area and cerebellum, and emphasize the participation of the cerebellum in the cognitive and emotional processing in humans (Schmahmann, 2004). Furthermore, it has also been demonstrated that the cerebellum plays a critical role in pure auditory processing involving the hemispheric portion of the right lobules I to VI (Petacchi et al., 2005). Consistent with these findings, we found that the right lobule VI presented declined FC with the lFPN, which might contribute to the dysfunction of the auditory and cognitive or emotional processing. Future studies should further explore cerebral-cerebellar loop pattern associated with auditory deprivation.

The insula is thought to be a brain area that facilitates the coordinated attention from different sensory modalities through the interactions of frontal-cingulate-parietal areas (Supekar and Menon, 2012; Chen et al., 2015). HL patients were indicated to develop attention deficits (Guerreiro and Van Gerven, 2017), and the altered FC in the insula with DAN might underlie the attention impairments caused by SNHL. The insula also plays an integrative role in the cortical circuits involving cognitive, emotional, and multi-sensory processing. Extensive connections were found between the insula and the auditory area and frontoparietal speech area (Shelley and Trimble, 2004), indicating the insula mediates the integration of auditory information and speech function. The anterior subdivision of the insula plays a crucial role in speech articulation and motor planning, and the posterior subdivision plays a crucial role in auditory speech processing (Dronkers, 1996), suggesting the insula contributes to the motor aspects of the speech production. Hence, the declined FC in the insula with SMN could be related to the multi-sensory integration and the perception or production of speech in the SNHL patients.

Furthermore, we observed significant correlations between the magnitudes of the functional coupling with apparent between-group differences in seed network to whole brain FC and anxiety state and episodic verbal memory, indicating that these functional abnormalities of seed network to whole brain FC might be involved in the neural mechanism of the possible emotional and cognitive dysfunction in SNHL.

Several limitations of this study must be noted. First, the current study was a cross-sectional design; more information about the dynamic alterations of the FC pattern with multiple RSNs could not be clarified. Further longitudinal studies are required to address this problem. Second, as a result of the limited sample size, we are unable to perform the subgroup assessment according to the cognitive or emotional states, and the severity or frequency characteristics of HL. Future studies with larger sample sizes may identify the functional abnormalities involved in the emotional or cognitive dysfuntion related to HL. Nevertheless, the sample size (30 SNHL patients and 39 controls) in the current study was similar to that of other fMRI studies on HL. Third, the head motion challenges the resting-state FC. To alleviate the impacts from head motion, we conducted several procedures. We excluded the participant whose head motion parameter exceeds a criterion. When computing the seed network to whole brain FC, the head motion covariance was regressed using a Friston-24 model. The motion metric was considered as a covariance when performing between-group comparisons. However, the influences of head motion still could not be entirely ruled out. Fourth, the neuropsychological tests were routine and rudimentary. Future research should further evaluate the specific cognitive or emotional functions related to HL. Fifth, the identification of the resting-state components depended on our selection. We selected a moderate model order of 40, which corresponds to that of previous studies (Schrepf et al., 2018). To validate the reliability, we also tried to identify the model order according to an automatic dimension estimation method. The estimated model order was 37, and the decompositions from both methods were similar. The 11 RSNs identified in this study could be recognized by both methods. Finally, we took advantage of multiple methods of analysis to reveal the network modifications in SNHL, which also posed a limitation to this study. We could only draw a general conclusion due to the large quantity of the results. We were unable to further analyze and discuss the specific role of each network alteration and their contributions to the specific function in SNHL in the current study. The specific mechanisms underlying those findings that we presented need be further addressed in the following work.

## Conclusion

This work sustained the functional reorganization involving multiple RSNs associated with higher-order cognitive, executive control, attention, visual, sensorimotor, and auditory functions following long-term bilateral SNHL. The dysconnectivity within and between the RSNs suggested these RSNs might contribute to the top–down control deficits, cross-modal reorganization, cognitive and emotional impairments associated with SNHL. The correlations between the RSN alterations and neuropsychological results shed lights on the neurophysiological mechanisms underlying the potential cognitive or emotional dysfunction that is driven by SNHL.

## Author Contributions

YL conducted the experiments, analyzed the data, and wrote the paper. CW helped integrate the data. YJ and TT helped with analyses of the data. JZ helped with the collection of the participants. GT directed this study, designed the research, and gave vital suggestions.

## Conflict of Interest Statement

The authors declare that the research was conducted in the absence of any commercial or financial relationships that could be construed as a potential conflict of interest.

## Footnotes

1 http://www.fil.ion.ucl.ac.uk/spm/

2 http://rfmri.org/dpabi/

3 http://icatb.sourceforge.net

4 http://www.restfmri.net/forum/

5 http://dbm.neuro.uni-jena.de/vbm

## Abbreviations

ACC: anterior cingulate cortex
aDMN: anterior default mode network
AN: auditory network
AVLT: Auditory Verbal Learning Test
BOLD: blood oxygenation level-dependent signal
CB: cerebellum network
DAN: dorsal attention network
DMN: default mode network
FC: functional connectivity
FDR: false discovery rate
FWHM: full-width half-maximum
GABA: gamma-aminobutyric acid
GLM: general linear model
GM: gray matter
GRF: Gaussian random field
HAM-D: Hamilton Depression Rating Scale
HL: hearing loss
ICs: independent components
ICA: independent component analysis
IPL: inferior parietal lobule
lFPN: left frontoparietal network
lobule VI: cerebellum lobule VI
LVN: lateral visual network
MMSE: Mini Mental State Examination
MNI: Montreal Neurological Institute
mPFC: medial prefrontal cortex
MRS: magnetic resonance spectroscopy
MVN: medial visual network
PCC: posterior cingulate cortex
pDMN: posterior default mode network
PET: Positron emission tomography
PMC: premotor cortex
pMTG: posterior middle temporal gyrus
pSTP: posterior superior temporal plane
pSTG/pSTS: posterior temporal gyrus/sulcus
pTMN: posterior temporal network
rFPN: right frontoparietal network
ROI: region of interest
Rs-fMRI: resting-state functional magnetic resonance imaging
RSNs: resting-state networks
SAS: Self-Rating Anxiety Scale
SDMT: Symbol Digit Modalities Test
SMN: sensory motor network
SNHL: sensorineural hearing loss
STG: superior temporal gyrus
VBM: voxel-based morphometry
vmPFC: ventromedial prefrontal cortex
VN: visual network
WM: white matter
